# Clinical and immunologic features of co-infection in COVID-19 patients, along with potential traditional Chinese medicine treatments

**DOI:** 10.3389/fimmu.2024.1357638

**Published:** 2024-03-21

**Authors:** Guochao Zhang, Junjun Zhang, Qi Gao, Yingying Zhao, Yanjun Lai

**Affiliations:** ^1^ Department of Clinical Laboratory, Ninth Hospital of Xi’an, Xi’an, Shannxi, China; ^2^ Xianyang Center for Disease Control and Prevention, Xianyang, Shannxi, China, China; ^3^ Department of Pathology, Fenyang College of Shanxi Medical University, Fenyang, Shanxi, China

**Keywords:** COVID-19, SARS-CoV-2, co-infection, immune cells, inflammatory factors

## Abstract

**Objectives:**

With the increasing number of people worldwide infected with SARS-CoV-2, the likelihood of co-infection and/or comorbidities is rising. The impact of these co-infections on the patient’s immune system remains unclear. This study aims to investigate the immunological characteristics of secondary infections in hospitalized COVID-19 patients, and preliminarily predict potential therapeutic effects of traditional Chinese medicine and their derivatives for the treatment of co-infections.

**Methods:**

In this retrospective cohort study, we included 131 hospitalized patients with laboratory-confirmed COVID-19, of whom there were 64 mild and 67 severe cases. We analyzed clinical characteristics and immunologic data, including circulating immune cell numbers, levels of inflammatory factors and viral load, comparing COVID-19 patients with and without co-infection.

**Results:**

Among 131 hospitalized COVID-19 patients, 41 (31.3%) were co-infection positive, with 33 (80.5%) having severe disease and 14 (34.1%) of them resulting in fatalities. Co-infected patients exhibited significantly higher severity and mortality rates compared to non-co-infected counterparts. Co-infected patients had significantly lower absolute counts of lymphocytes, total T lymphocytes, CD4^+^ T cells, CD8^+^ T cells and B lymphocytes, while levels of hs-CRP, PCT and IL-6 were significantly elevated compared to non-co-infected patients. Additionally, the viral load of co-infected patients was significantly higher than non-co-infected patients.

**Conclusion:**

Co-infection emerges as a dangerous factor for COVID-19 patients, elevating the risk of severe pneumonia and mortality. Co-infection suppresses the host’s immune response by reducing the number of lymphocytes and increasing inflammation, thereby diminishing the antiviral and anti-infective effects of the immune system, which promotes the severity of the disease. Therefore, it is crucial to implement infection prevention measures to minimize the spread of co-infections among COVID-19 hospitalized patients. Additionally, changes in these biomarkers provide a theoretical basis for the effective treatment of co-infections with traditional Chinese medicine.

## Introduction

The coronavirus disease 2019 (COVID-19) is a highly infectious respiratory disease caused by Severe Acute Respiratory Syndrome Coronavirus 2 (SARS-CoV-2), which belongs to RNA beta coronavirus family. Since its emergence in 2019, COVID-19 has rapidly spread globally, posing significant threats to both human health and the global economy (1, 2). As of 27 August 2023, there have been over 770 million confirmed cases and 6.9 million deaths reported globally. The number of SARS-CoV-2 infections is increasing by more than 50,000 per day, and it is estimated that a large number of cases have not been reported (3, 4). While efforts to understand COVID-19, including its origin, pathogenesis and therapeutic strategies, are ongoing, the development of effective antiviral vaccines has been a crucial milestone in combating the epidemic. Currently, more than 200 traditional or novel vaccines are in development, with some already widely deployed in several countries (5–8). Global vaccination efforts have significantly reduced the prevalence, severity and mortality of COVID-19. However, the emergence of SARS-CoV-2 mutant strains poses a significant challenge to the effectiveness of vaccines (9, 10). As SARS-CoV-2 variants increasingly evade neutralizing antibody responses, cellular immune responses are gaining importance in the immunological context.

At present, specific antiviral agents for COVID-19 are still under study. α-Interferons, ribavirin, lopinavir/ritonavir, etc., are only approved for emergency use, and their distribution is controlled. In contrast, antibiotics are easily used to treat secondary bacterial infections. However, the empirical or preventive use of antibiotics can easily lead to the development of drug-resistant bacteria. In addition, critically ill patients, requiring respiratory support and intensive care, are particularly susceptible to secondary and opportunistic infections, including co-infections with other pathogens such as influenza viruses (11). Early studies have indicated a direct association between bacterial and/or viral co-infections and more severe outcomes during pandemics (12, 13). Bacterial infections, especially *streptococcus pneumonia, staphylococcus aureus* and fungal, are common complications of influenza-induced pneumonia, raising questions whether co-infection exacerbates the development of severe COVID-19 and how to influence the disease’s progression (14). In this context, the development of new antibacterial and antiviral drugs is a global priority to meet the ongoing demand caused by COVID-19 and reduce the number of deaths associated with bacterial infections worldwide. Traditional Chinese medicine, primarily derived from plants and with a long history as sources of therapeutic agents, offers several advantages, including multiple targets, potential efficacy, and minimal toxic side effects. Research has shown that traditional Chinese medicine can lower the incidence of serious or critical events, improve clinical recovery rates, and help alleviate symptoms such as cough or fever (15). Artemisinin, one of the latest and most successful natural products, along with its derivatives, has served as a first-line antimalarial drug in dozens of countries. Compared to other antimalarial drugs, artemisinin can rapidly reduce parasitic diseases and delay drug resistance when used in combination therapy (16). Additionally, artemisinin has shown positive effects in treating malaria, cancer, viral infections, bacterial infections, and some cardiovascular diseases (17). Traditional Chinese medicine and their derivatives may also produce unexpected positive effects in the treatment of COVID-19 and the management of co-infections.

The role of the immune system in the onset and progression of co-infections in COVID-19, particularly its link to symptom severity, remains unclear. Several studies have reported immune system impairments induced by co-infections in COVID-19 (11, 13, 18–20). In co-infections in COVID-19 cases, patients often exhibit lymphopenia and elevated levels of various cytokines and chemokines, leading to a hyperinflammatory response known as a cytokine storm. To elucidate the immune response and pathogenesis of co-infections in COVID-19 patients, we conducted a study involving 131 COVID-19 patients, comparing cellular immune and inflammatory factor differences between those with and without co-infections. These findings may help us broaden our understanding of the risk factors associated with disease severity in co-infection among COVID-19 patients. Additionally, this provides a theoretical basis for the development of traditional Chinese medicine and their derivatives to effectively treat co-infections.

## Materials and methods

### Study design and patients

The research is a retrospective cohort study. We selected a total of 131 hospitalized patients who had been admitted to the hospital and were confirmed with COVID-19 between December 17, 2022 to January 28, 2023, in the Ninth Hospital of Xi’an (Shaanxi, China). The diagnosis and staging of COVID-19 were determined based on the diagnosis and treatment scheme of COVID-19 (9th edition) issued by the National Health Commission of China (21). Specifically, patients were diagnosed with COVID-19 if they were tested positive for SARS-CoV-2 viral RNA and exhibited clinical symptoms such as fever and cough. Patients diagnosed with COVID-19 were categorized as asymptomatic, mild, moderate, severe and critical cases based on the severity of their symptoms. In this study, we included patients with both mild and severe symptoms. Mild patients encompassed those with moderate symptoms, while severe patients encompassed those with critical symptoms. According to whether these patients were co-infected, they were categorized into non-co-infected and co-infected groups, excluding asymptomatic patients with COVID-19. Clinical characteristics and laboratory data of the patients were obtained from medical records. The data were examined by two physicians, and a third researcher ruled any differences in interpretation between the two primary reviewers. The study was performed in accordance with Good Clinical Practice and the Declaration of Helsinki principles for ethical research. The study complied with all relevant ethical regulations and the protocol was approved by the institutional review board of the Ninth Hospital of Xi’an (NO.202311), and a waiver of informed consent was granted, due to the retrospective nature of the observational design.

### Clinical classifications

According to the guidelines for the diagnosis and management of COVID-19 (9th edition, in Chinese) released by the National Health Commission of China (21), the clinical classifications of COVID-19 are as follows. Mild cases: Clinical symptoms are mild, and there are no signs of pneumonia on imaging. Moderate cases: Patients exhibit symptoms such as fever and respiratory tract symptoms, along with pneumonia manifestation visible in imaging. Severe cases: Meet any of the following criteria — respiratory distress with a respiratory rate ≥ 30 breaths/min; SpO_2_ ≤ 93% at rest; and PaO_2_/FIO_2_ ≤ 300. Patients with over 50% lesion progression within 24 to 48 hours in pulmonary imaging should be treated as severe cases. Critical cases: Those meeting one of the following conditions — respiratory failure requiring mechanical ventilation, shock, or complications from other organ failures necessitating monitoring and treatment in the ICU.

### Real time PCR for quantification of SARS-CoV-2 viral load

Pharyngeal swabs were collected and stored at -20 °C. Viral RNA was extracted following the manufacturer’s instructions (Daan Genetics Co., Ltd., China). Subsequently, 20μL of RNA was mixed with a commercial real-time PCR master mix (Daan Gene Co., Ltd. Guangzhou, China) in a 20μl reaction containing primers. The detection limit of the ORF1ab/N real-time reverse transcription polymerase chain reaction (RT-PCR) assays was approximately 200 copies per milliliter. Samples with cycle threshold (Ct) values of ≤40.0 were considered positive for SARS-CoV-2 RNA. RT-PCR analyses for all assays were performed on the ABI 7500 real-time PCR system (Applied Biosystems, USA).

### Lymphocyte phenotyping by flow cytometry

We collected peripheral blood from patients using EDTA anticoagulation tubes. Briefly, antibodies were added to whole blood, and samples were lysed with FACS lysing solution (Beckman Coulter, Indianapolis, IN, USA). Peripheral blood mononuclear cells (PBMC) were isolated, and subpopulations were detected by flow cytometry. All experimental procedures were conducted strictly in accordance with the instructions provided for the reagents. Antibodies were purchased from Beckman Coulter Inc. T cell were labeled using anti-CD45-FITC/anti-CD4-PE/anti-CD8-ECD/anti-CD3-PC5.5. B and NK cells were labeled using anti-CD45-FITC/anti-CD56-PE/anti-CD19-ECD/anti-CD3-PC5.5/anti-CD16-PE. Kaluza flow cytometry software version 2.1 (Beckman Coulter, Indianapolis, IN, USA) was used for data analysis.

### Measurement of levels of serum hs-CRP, PCT and IL-6

All blood samples for initial high-sensitivity C-reactive protein (hs-CRP), procalcitonin (PCT) and IL-6 measurements were obtained within ≥ 48h of the clinical diagnosis of COVID-19. Whole blood was collected in serum-separating tubes. Serum biomarker levels were measured in duplicate in samples kept on ice prior to measurement. Inflammatory factors, including hs-CRP, PCT and IL-6, were tested by Siemens Immulite 1000 and Roche Cobas c411 biochemical analyzer chemiluminescence immunoassay (CLIA).

### Collection of sputum samples and microbial culture

Consecutive deep sputum specimens were collected from patients with COVID-19. Subsequently, 10 μL of sputum specimen was inoculated in Columbia blood agar, Chocolate agar plus bacitracin, and chocolate agar, and incubated in air plus 5% carbon dioxide (37 °C, 48 h). Individual colonies were picked and prepared for testing. Isolates were identified by matrix assisted laser desorption ionization time-of-flight (MALDI-TOF) mass spectrometry (Bruker Daltonics) and, where necessary, appropriate API kits (bioMérieux).

### Statistical analyses

Statistical analyses were performed using Graphpad Prism version 9.5.0 (GraphPad, La Jolla, CA, USA). Student’s t test was performed for two-group analysis. The relationship between 2 variables was examined by simple linear regression analysis. For analysis of contingency tables, Fisher’s exact or χ^2^ test was used. A *p*-value < 0.05 was considered statistically significant.

## Results

### Basic information of patients with COVID-19

In order to understand the immune response and clinical characteristics of COVID-19 patients, 131 COVID-19 patients were included in this retrospective cohort study. All of them were admitted to the Ninth Hospital of Xi’an and underwent CT scanning and SARS-CoV-2 viral RNA testing, with positive results confirming SARS-CoV-2 infection after admission. Among the participants, 64 patients were mild cases, and 67 patients were severe cases. The clinicopathologic characteristics of these patients were detailed in **Table 1**. 23 patients died during hospitalization, and 118 were discharged from the hospital. The gender distribution of mild cases is balanced, while in severe cases, the proportion of males is significantly higher than females. The average age of severe cases was significantly higher than that of mild cases. Severe cases took an average of 26.40 days from symptoms onset to discharge (deceased patients were excluded) which was significantly longer than the 17.84 days for mild cases. Most patients had underlying health conditions, with hypertension being the most common complication. Severe cases had a higher frequency of underlying diseases compared to mild cases in the context of COVID-19. It is noteworthy that Alzheimer’s disease (5 cases) and Parkinson’s disease (4 cases) were present among severe cases, while they were difficult to be detected in mild cases. Among severe patients, there were 23 deceased individuals, including 6 females, a significantly lower number compared to the 17 male deaths. The average age of deceased patients was over 60 years old, with 14 patients being older than 80.

**Table 1:** Demographics and clinical characteristics of patients with COVID-19.

	Mild cases (n=64)	Severe cases (n=67)	*P*
Females (%)	36 (56.3)	20 (29.9)	0.002
Males (%)	28(43.7)	47(70.01)	0.002
Age, mean, years (± SD)	71.14 (11.82)	78.57 (9.444)	0.0001
Days since symptoms onset (± SD)	17.84 (4.42)	26.40 (7.87)	<0.0001
Underlying diseases			
Chronic lung disease (%)	12 (18.8)	14 (20.9)	0.758
Chronic heart disease (%)	23 (35.9)	33 (49.3)	0.124
Chronic liver failure (%)	5 (7.8)	7 (10.4)	0.601
Chronic kidney disease (%)	1 (1.6)	5 (7.5)	0.106
Alzheimer’s disease (%)	0 (0)	5 (7.5)	
Parkinson’s disease (%)	0 (0)	4 (6.0)	
Type 2 diabetes (%)	11 (17.2)	19 (28.4)	0.128
Hypertension (%)	34 (53.1)	42 (62.7)	0.268
Bacterial superinfection (%)	8 (12.5)	33 (49.3)	<0.0001
Females/males who died (%)	0 (0)	6 (26)/17 (74)	
Deaths over 80 years old (%)	0 (0)	14 (61)	

Results are expressed as mean ± standard deviation (SD) or percentage; *P* values are from a χ^2^ analysis.

During medical treatment, we found that out of the 131 patients with COVID-19, 41(31.3%) were co-infection positive and 90 were co-infection negative. Among the 90 non-co-infected patients, 56 (62.2%) were mild cases, and 34 (37.8%) were severe cases. Among the 41 co-infected patients, there were 8 (19.5%) mild cases and 33 (80.5%) severe cases. These results indicated that co-infected patients faced a significantly higher risk of developing severe symptoms. The mean duration from symptom onset to discharge was significantly longer in co-infected patients (27.07 d) compared to non-co-infected patients (19.33 d). Additionally, it was observed that 9 (10%) of the non-co-infected patients passed away, whereas 14 (34.1%) deaths occurred among co-infected patients, demonstrating a significantly higher mortality rate in this group. Among the 33 severe patients with co-infections, 18 (54.5%) had single bacterial infections, 15 (45.5%) mixed bacterial infections. In contrast, all 8 mild patients with co-infections were infected with single bacterial strains, and no mixed bacterial infections were observed. Furthermore, 18 cases with fungal infections and 14 cases with Acinetobacter baumannii were detected in severe patients with co-infections. In mild patients with co-infections, only 1 case of fungal infection was found, and Acinetobacter baumannii was difficult to detect. Notably, while isolating pathogenic bacteria from severe cases, we found one patient infected with carbapenem-resistant Pseudomonas aeruginosa (CRPA), six patients with carbapenem-resistant Acinetobacter baumannii (CRAB) and one patient with carbapenem-resistant enterobacteriaceae (CRE) infection. No resistant organisms were found in the mild cases. The basic information of patients with coinfections was presented in **Table 2**.

**Table 2:** Epidemiology of pathogen co-infections at COVID-19 admission.

	Mild cases (n=8)	Severe cases (n=33)	*P*
Single pathogen infection (%)	8 (100)	18 (54.5)	
Mixed pathogen infection (%)	0 (0)	15 (45.5)	
Fungal infection (%)	1 (12.5)	18 (54.5)	0.032
*Candida albicans* (%)	1 (12.5)	14 (42.4)	0.115
*Aspergillus fumigatus* (%)	0 (0)	4 (12.1)	
*Staphylococcus aureus* (%) (G^+^)	2 (25)	2 (6.1)	0.105
*Streptococcus pneumoniae* (%) (G^+^)	1 (12.5)	2 (6.1)	0.530
*Enterococcus faecalis* (%) (G^+^)	0 (0)	1 (3.0)	
*Staphylococcus hominis* (%) (G^+^)	0 (0)	1 (3.0)	
*Pseudomonas aeruginosa* (G^-^)	1 (12.5)	4 (12.1)	0.977
*Acinetobacter baumannii* (G^-^)	0 (0)	14 (42.4)	
*Klebsiella pneumoniae* (G^-^)	2 (25)	5 (15.2)	0.507
*Legionella pneumophila* (G^-^)	1 (12.5)	0 (0)	
*Enterobacter asburiae* (G^-^)	0 (0)	1 (3.0)	
*Stenotrophomonas maltophilia* (G^-^)	0 (0)	3 (9.1)	
*Escherichia coli* (G^-^)	0 (0)	1 (3.0)	
*Enterobacter cloacae* (G^-^)	0 (0)	2 (6.1)	
*Mycoplasma pneumoniae*	0 (0)	4 (12.1)	
CR-PA	0 (0)	1 (3.0)	
CR-AB	0 (0)	6 (18.2)	
CRE	0 (0)	1 (3.0)	
No. Death	0 (0)	14 (42.4)	

Results are expressed as mean ± standard deviation (SD) or percentage; *P* values are from a χ2 analysis. G+, Gram positive; G-, Gram negative; CRPA, carbapenem-resistant Pseudomonas aeruginosa; CRAB, carbapenem-resistant Acinetobacter baumannii; CRE, carbapenem-resistant Enterobacteriaceae.

### Measurement of immune cells in COVID-19 patients with co-infection

**Figure 1:**
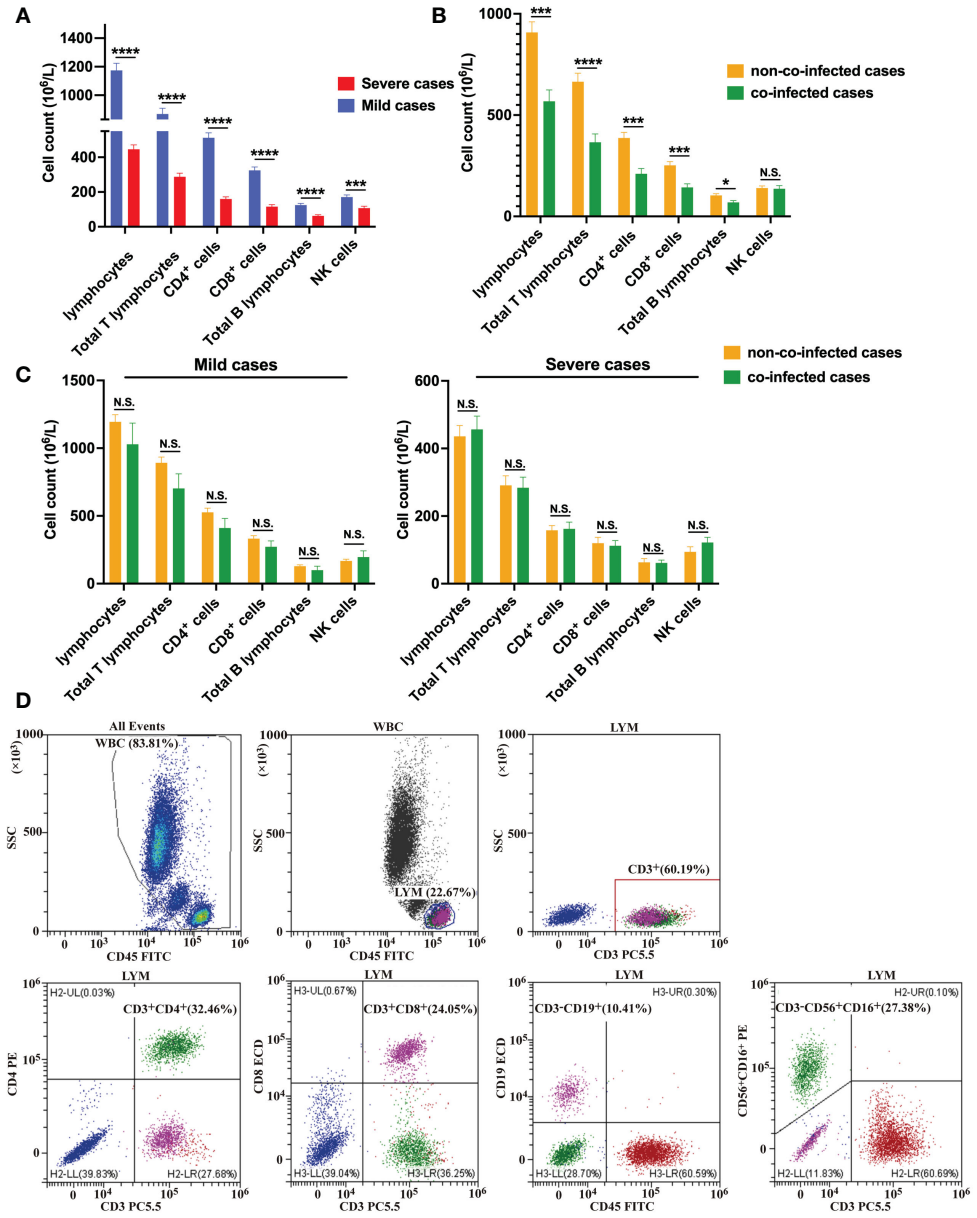
Immune cell counts in COVID-19 patients. **(A)** Comparisons of the absolute number of immune cells between severe cases (n = 67) and mild cases (n = 64). **(B)** Comparisons of the absolute number of immune cells between co-infected cases (n = 41) and non-co-infected cases (n = 90). **(C)** Comparisons of the absolute number of immune cells between co-infected and non-co-infected cases based on disease severity. **(D)** Flow cytometric analysis of lymphocytes, total B lymphocytes, NK cells, total T lymphocytes, CD4^+^ T cells and CD8^+^ T cells from a representative patient. All data presented as the mean ± SEM. Differences were tested using unpaired *t* test. *p*<0.05 was considered significant. *p<0.05, **p<0.01, ***p<0.001, ****p<0.0001; N.S., not significant.

We also conducted detection of circulating immune cells in 131 patients with COVID-19, and the results are presented in **Table 3**. Preliminary analysis of circulating immune cells revealed that the counts of Lymphocytes, total T lymphocytes, CD4^+^ T cells, CD8^+^ T cells, B cells and NK cells in mild cases were within the normal reference range and near the lower limit of reference values. In severe cases, NK cell counts were within the normal reference range, while all other types of immune cell counts were below the normal reference range. In severe cases, the number of lymphocytes, total T lymphocytes, CD4^+^ T cells, CD8^+^ T cells and B lymphocytes decreased significantly compared to mild cases, as shown in **Figure 1A**. The assay strategy was illustrated in **Figure 1D**. The proportion of total T lymphocytes, CD4^+^ T cells in severe cases was significantly lower than in mild cases, whereas the proportion of B cells and NK cells was significantly higher than in mild cases. This difference may be attributed to the more significant reduction of T cells in the severe cases. There was no significant difference in the proportion of CD8^+^ T cells between mild and severe cases.

**Table 3:** Immunological features of patients with COVID-19.

	reference range	Mild cases (n=64)	Severe cases (n=67)	*P*	non-co-infected cases (n=90)	co-infected cases (n=41)	*P*
Lymphocytes ×10^6^/L (± SD)	1100 - 3200	1174 (399.3)	446 (205.5)	<0.0001	907.9 (495.2)	568.3 (357.1)	0.0001
Total T cell count, ×10^6^/L (± SD)	723 - 2271	868.1 (323.6)	287.4 (171.1)	<0.0001	664.8 (399.7)	365.6 (265.8)	<0.0001
Total T cells, % (± SD)	61.1 - 77	73.57 (9.29)	62.14 (17.56)	<0.0001	71.06 (13.27)	60.38 (16.70)	0.0001
CD4^+^ T cell count, ×10^6^/L (± SD)	396 - 1309	511.8 (230.3)	159.8 (99.33)	<0.0001	387.0 (261.1)	210.5 (166.0)	0.0001
CD4^+^ T cells, % (± SD)	25.8 - 41.6	42.92 (10.59)	35.36 (13.16)	0.0004	41.05 (11.79)	34.65 (13.08)	0.0062
CD8^+^ T cell count, ×10^6^/L (± SD)	224 - 1014	324.6 (155.4)	115.9 (95.03)	<0.0001	251.9 (173.8)	143.2 (114.2)	0.0004
CD8^+^ T cells, % (± SD)	18.1 - 29.6	28.16 (10.33)	24.31 (13.31)	0.0676	27.30 (12.57)	23.76 (10.58)	0.1192
B cell count, ×10^6^/L (± SD)	118 - 645	124.8 (78.79)	62.31 (56.05)	<0.0001	103.7 (79.67)	69.00 (56.41)	0.0132
B cells, % (± SD)	9.02 - 14.10	10.55 (6.086)	14.10 (12.78)	0.0458	11.71 (8.113)	13.81 (13.74)	0.2752
NK cell count, ×10^6^/L (± SD)	61 - 607	171.1 (98.05)	107.7 (89.51)	0.0002	139.8 (98.11)	136.2 (101.2)	0.8466
NK cells, % (± SD)	10.04 - 19.78	14.52 (7.92)	21.72 (13.67)	0.0004	15.90 (10.09)	23.26 (13.59)	0.0007
CRP, mg/L (± SD)	0 - 3	20.80 (13.51)	121.4 (64.03)	<0.0001	49.80 (54.45)	121.6 (71.51)	<0.0001
PCT, ng/mL (± SD)	0 - 0.046	0.08 (0.11)	1.26 (1.34)	<0.0001	0.3223 (0.7042)	1.470 (1.446)	<0.0001
IL-6, pg/mL (± SD)	0 - 7	19.58 (12.63)	102.8 (77.13)	<0.0001	42.27 (46.35)	105.7 (90.11)	<0.0001

Results are expressed as mean ± standard deviation (SD) or percentage; *P* values are from a unpaired t test. CRP, C-reactive protein; PCT, procalcitonin.

The numbers of lymphocytes, total T lymphocytes, CD4^+^ T cells, CD8^+^ T cells, and B lymphocytes were significantly lower in co-infected patients than those in non-co-infected patients, and there was no difference in the number of NK cells between the two groups, as is shown in **Figure 1B**. The proportion of total T lymphocytes, CD4^+^ T cells in co-infected patients were significantly lower than those in non-co-infected patients, while the proportion of NK cells in co-infected patients was significantly higher than that in non-co-infected patients. The proportion of CD8^+^ T cells and B cells did not differ significantly between the two groups. Additionally, we found that among mild cases, the number of lymphocytes, total T lymphocytes, CD4^+^ T cells, CD8^+^ T cells and B lymphocytes in co-infected patients were slightly lower than those in non-co-infected patients. However, there was no significant difference between the groups. Among severe cases, there was no difference in circulating immune cell counts between the groups, as is shown in **Figure 1C**.

### SARS-CoV-2 viral load in COVID-19 patients with co-infection

**Figure 2:**
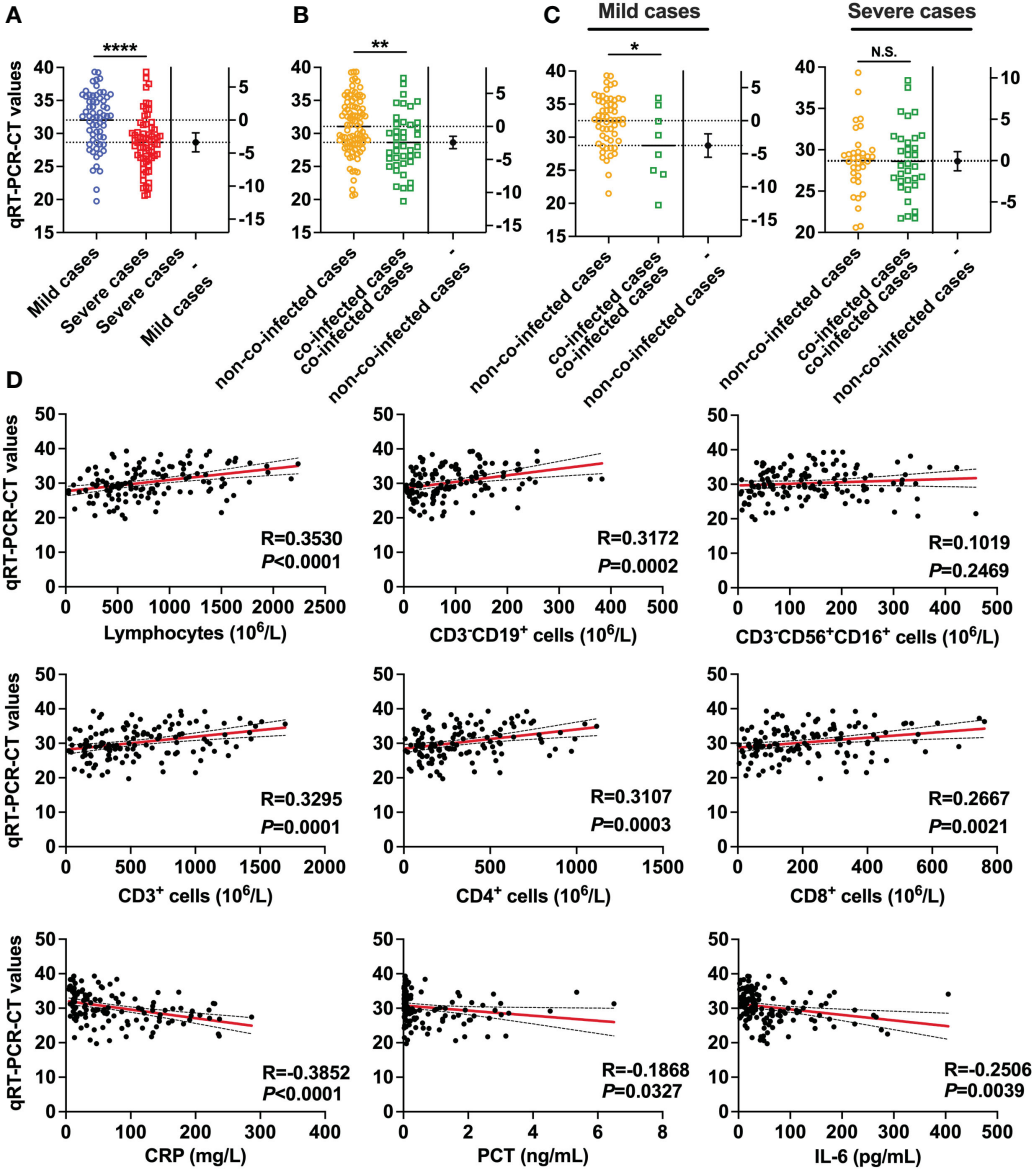
SARS-CoV-2 viral RNA RT-PCR CT values in patients with COVID-19. **(A)** Comparisons of SARS-CoV-2 viral RNA RT-PCR-CT values between severe cases (n = 67) and mild cases (n = 64). **(B)** Comparison of SARS-CoV-2 viral RNA RT-PCR-CT values between co-infected cases (n = 41) and non-co-infected cases (n = 90). **(C)** Comparison of SARS-CoV-2 viral RNA RT-PCR-CT values between co-infected cases and non-co-infected cases by disease severity. **(D)** Correlations of SARS-CoV-2 viral RNA RT-PCR-CT values with the number of Lymphocyte, Total B lymphocyte, NK cell, total T lymphocytes, CD4^+^ T cells, and CD8^+^ T cells, as well as the levels of hs-CRP, PCT and IL-6. All data are presented as the mean ± SEM. Differences were tested using unpaired t test. *p*<0.05 was considered significant. *p<0.05, **p<0.01, ***p<0.001, ****p<0.0001; N.S., not significant.

To assess the relationship between COVID-19 patient severity and SARS-CoV-2 viral load, we conducted tests on SARS-CoV-2 viral loads in throat swab samples from all patients. The results revealed that patients with higher SARS-CoV-2 viral loads were more prone to developing severe pneumonia. Specifically, the SARS-CoV-2 viral RNA RT-PCR CT value for severe patients was 28.65, significantly lower than that for mild patients (32.03). A smaller CT value in viral nucleic acid detection indicates a higher viral load, as demonstrated in **Figure 2A**. Moreover, the viral load of co-infected patients (CT value = 28.64) was significantly higher than that of non-co-infected patients (CT value = 31.06), as indicated in **Figure 2B**. In mild patients, the viral load of co-infected patients was significantly elevated compared to non-co-infected patients. However, among severe patients, there was no notable difference in viral load between the groups, as shown in **Figure 2C**. To explore the impact of SARS-CoV-2 viral load on circulating immune cells and changes in inflammatory factors, we conducted a correlation analysis, the results of which are presented in **Figure 2D**. With the increase in SARS-CoV-2 viral load, the counts of lymphocytes, total T lymphocytes, CD4^+^ T cells, CD8^+^ T cells and B lymphocytes in patients decreased, indicating a negative correlation. Additionally, serum levels of hs-CRP, PCT and IL-6 were elevated, showing a positive correlation. Importantly, there was no significant correlation between the number of NK cells and SARS-CoV-2 viral load.

### The levels of hs-CRP, PCT and IL-6 levels were measured in COVID-19 patients with co-infection

**Figure 3:**
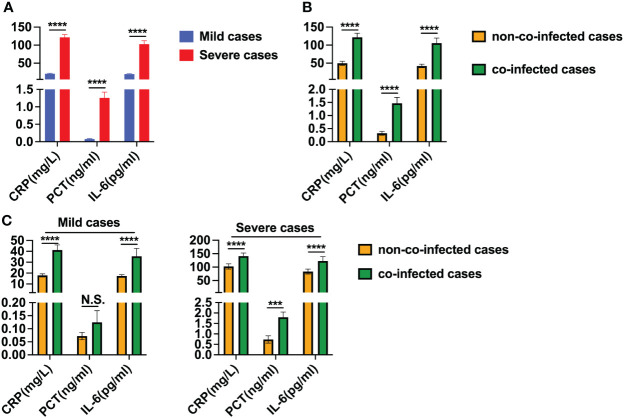
Levels of hs-CRP, PCT and IL-6 in patients with COVID-19. **(A)** Comparison of hs-CRP, PCT and IL-6 levels between severe cases (n = 67) and mild cases (n = 64). **(B)** Comparison of hs-CRP, PCT and IL-6 levels between co-infected cases (n = 41) and non-co-infected cases (n = 90). **(C)** Comparison of hs-CRP, PCT and IL-6 levels between co-infected and non-co-infected cases based on disease severity. All data are presented as the mean ± SEM. Differences were tested using unpaired t test. *p*<0.05 was considered significant. *p<0.05, **p<0.01, ***p<0.001, ****p<0.0001; N.S., not significant.

Serum inflammatory cytokines were assessed in 131 patients with COVID-19 to determine the presence of a cytokine storm. We observed that hs-CRP, PCT and IL-6 levels were higher than the upper limit of the normal reference range in nearly all mild and severe patients, as detailed in **Table 3**. In severe patients, hs-CRP, PCT and IL-6 levels were significantly higher than those in mild patients, as depicted in **Figure 3A**. Moreover, in co-infected patients, hs-CRP, PCT and IL-6 levels were significantly elevated compared to non-co-infected patients, as shown in **Figure 3B**. In mild patients, hs-CRP and IL-6 were significantly increased in co-infected patients compared to non-co-infected patients, while PCT showed no significant increase. Conversely, in severe patients, hs-CRP, PCT and IL-6 levels in co-infected patients were significantly higher than those in non-co-infected patients, as illustrated in **Figure 3C**.

## Discussion

COVID-19 has resulted in 6.9 million deaths worldwide. A recent meta-analysis examined the impact of tuberculosis on the severity of COVID-19 and found that SARS-CoV-2 and *Mycobacterium tuberculosis* were the primary causes of death from infectious diseases. Simultaneously, the mortality rate increased among patients diagnosed with a co-infection of COVID-19 and tuberculosis (20). Another meta-analysis explored viral co-infections in COVID-19, suggesting that such co-infections may exacerbate the severity of COVID-19 and influence treatment and prevention strategies (11). However, hospitalized patients with COVID-19 are vulnerable to hospital-acquired infections, and those with co-infections face a significantly increased risk of mortality. A study revealed that 94.2% of COVID-19 patients were concurrently infected with various other microorganisms, including viruses, bacteria and fungi (22). In this study, we analyzed a total of 131 patients with COVID-19, among whom 41 were co-infected. This co-infection rate of 31.3% is significantly higher than what has been reported in previous studies (23). Compared to patients with COVID-19 alone, those with co-infections faced a 3.5 times higher risk of death. Males and older patients, especially those with underlying comorbidities such as Alzheimer’s disease, Parkinson’s disease and bacterial infections, were at increased risk of developing severe pneumonia. Interestingly, co-infection was rare in mild cases, whereas in severe cases, the incidence of co-infection was significantly higher. Co-infection may easily develop into severe disease. Furthermore, severe patients exhibited a significant increase in the proportion of mixed bacterial infections, fungal infections and drug-resistant bacterial infections which could be attributed to prolonged antibiotic use and nosocomial infections resulting from mechanical ventilation. These factors may play a crucial role in the deterioration and, in some cases, fatalities observed in co-infected patients.

As SARS-CoV-2 variants increasingly evade neutralizing antibody responses, cellular immune responses are gaining importance in the immunological context. During the diagnosis and treatment of COVID-19, the presence of SARS-CoV-2 alongside other respiratory pathogens may result in overlapping clinical symptoms, making it challenging for clinicians to exclude other pathogens from the infection. Co-infected patients more frequently experience respiratory distress and may develop acute respiratory distress syndrome. In terms of laboratory findings, lymphopenia is more common in co-infected cases than in non-co-infected patients. Research has found that the interaction between SARS-CoV-2 and Mycobacterium tuberculosis co-infection can modulate immune responses, particularly affecting monocytes, macrophages, neutrophils and T cells, resulting in weakened immunity and increased inflammation (20). Sharov et al. (19) discovered that COVID-19 led to a rapid increase in the depletion of T cells, initially caused by HIV. The numbers of CD4^+^ and CD8^+^ cells decreased, accompanied by a sharp rise in the number of exhausted T cell, contributing to the deterioration of COVID-19-related respiratory distress. These findings are consistent with our research. Co-infection can suppress the antiviral effect of the immune system by reducing the number of T lymphocytes. In the majority of patients with severe COVID-19 disease, decreased T-lymphocyte counts are observed, whereas only a small proportion of patients with mild disease show decreased T-lymphocyte counts. This may be attributed to the increased expression of apoptosis factors FAS and FAS-L in T cells, B cells and NK cells. Additionally, research has found that high expression of PD-1 and TIM-3 genes on CD4^+^ and CD8^+^ T cells leads to T cell depletion and dysregulation of inflammatory cytokine secretion (24). Specifically, CD4^+^ T lymphocytes serve as clear indicators of impaired immune function in patients with COVID-19 and hold predictive value for the disease’s severity, with their counts correlating with the seriousness of the illness (25). CD8^+^ T lymphocytes, as effector T cells that specifically kill target cells, have the capability to inhibit and counteract viruses by releasing various cytokines to eliminate target cells. Notably, changes in CD8^+^ T lymphocytes are not apparent in the early stage of SARS-CoV-2 infection when the condition is mild. However, in severe conditions, cellular immune function becomes significantly impaired, leading to a decrease in CD8^+^ T lymphocytes (26). Generally, co-infection, caused by the combined action of one or more pathogens, can lead to reduced lymphocyte counts, overexpression of inflammatory factors, immune suppression in the host, resistance to antibiotics, and overall poor prognosis (11, 18). Our findings revealed that co-infection significantly reduces the number of circulating immune cells and lymphopenia being more common in co-infected than in non-co-infected patients. In co-infected COVID-19 patients, NK cell counts were within the normal reference range, but other immune cell counts were significantly lower than those in non-co-infected patients. This decline in circulating immune cells made it challenging for adaptive immunity to exert antiviral effects, thereby facilitating mild cases progression to severe ones.

Real-Time PCR-based detection of viral nucleic acids is the gold standard for diagnosing COVID-19. This technique allows for the immediate and easy determination of viral load values, indirectly indicating the severity of the infection. Consistent with previous studies (27, 28), we observed variations in viral load among patients with different disease severities. Severe patients exhibited significantly higher viral loads compared to mild patients, suggesting that a high viral load might be a risk factor for severe disease. Older patients with more comorbidities had higher SARS-CoV-2 viral loads. In addition, in mild patients, viral loads were significantly higher in patients with co-infections. This could be attributed to the compromised immune system and diminished ability to fight the virus in older, frail individuals. Another contributing factor could be the higher levels of angiotensin-converting enzyme 2 in the alveoli of the elderly, which is considered a receptor for SARS-CoV-2 (29). These factors lead to increased viral loads and more severe disease upon admission. We also discovered an association between viral load and an elevated risk of systemic inflammation, disease progression and death. Increased viral load due to co-infection led to a decrease in the number of circulating immune cells in patients. Simultaneously, levels of inflammatory factors rose, indicating a hyperinflammatory state. Viral load negatively correlated with the count of peripheral blood lymphocytes, total T lymphocytes, CD4^+^ T cells and CD8^+^ T cells, while showing positive correlations with inflammatory markers such as hs-CRP, PCT and IL-6. These findings suggest that viral load, circulating immune cell count, and serum inflammatory factor concentrations play pivotal roles in the progression of COVID-19 patients and contribute to risk stratification among hospitalized COVID-19 patients. This information could aid in predicting poor prognoses.

In the COVID-19 disease process, the inflammatory response plays a pivotal role and an inflammatory cytokine storm escalates the severity of the condition (30). Infection-related biomarkers such as hs-CRP, PCT and IL-6 levels are commonly utilized laboratory indicators in diagnosing and treating infectious lung diseases. In our study, we observed elevated serum levels of hs-CRP, PCT and IL-6 in both mild and severe COVID-19 cases, with significantly higher levels in severe cases than in mild ones. These findings align with previous research emphasizing the pivotal role of inflammatory factors in the progression from mild to severe disease (31). Xia et al. (32) found that serum procalcitonin levels were significantly higher in children with COVID-19 compared to those with other common respiratory infections. This suggests that co-infection with other pathogens may contribute to the increased inflammatory response. Pink et al. (33) discovered that approximately 32% of COVID-19 patients developed secondary bacterial infections during hospitalization. Infected patients with secondary bacterial infections had significantly higher PCT and hs-CRP levels compared to their initial admission. Co-infected patients exhibited notably elevated levels of hs-CRP, PCT and IL-6. It can be inferred that hs-CRP, PCT, and IL-6 assays could assist in identifying co-infected patients, especially in severe cases. Co-infection can activate inflammatory factors, potentially exacerbating the severity of the disease. Hence, the severity of COVID-19 disease can be assessed based on the level of inflammatory factors.

During China’s fight against COVID-19, traditional Chinese medicine has demonstrated significant benefits in alleviating symptoms and preventing disease deterioration. Moreover, it has proven effective in combating the spread of infectious diseases. Based on laboratory test results of co-infected patients, we speculate that traditional Chinese medicine or extracts with antiviral, antibacterial, anti-inflammatory, and immune system-improving properties have a positive effect on the treatment of co-infected patients. Honeysuckle, used in China for thousands of years, has been an effective treatment for viral infections. It can effectively inhibit virus replication *in vitro* and *in vivo*. Li M et al. (34) discovered that the Honeysuckle acids-flavonoids mixture has broad-spectrum antiviral activity and can significantly inhibit H1N1 and H3N2 influenza viruses. Additionally, honeysuckle can reduce IL-6 or TNF-α release induced by the SARS-CoV-2, inhibiting cytokine storm and virus replication by blocking the binding of the SARS-CoV-2 spike protein to the ACE2 receptor to form a syncytium (35). Research has found that honeysuckle and its extract chlorogenic acid have antibacterial and immune-enhancing effects, and can inhibit the production of multidrug-resistant bacteria by inhibiting protein synthesis (36–38). Therefore, honeysuckle and its derivatives are expected to bring good therapeutic effects to co-infected patients. Berberine, Matrine, Ginkgo Biloba Extract, Allicin, Ginsenosides, etc., are also widely used in traditional Chinese medicine for antibacterial and antimicrobial treatment (39–43). They have been validated to some extent in modern pharmacological research and show potential therapeutic effects on co-infections.

Our study has several limitations, as well. Firstly, this was a single-center observational cohort study, and the patient data were exclusively collected from northwest China. The sample quantity was not big enough, particularly for mild cases with co-infections. Therefore, it is crucial to interpret the results cautiously, as statistical insignificance might not rule out differences between cases of COVID-19 and co-infections in mild patients. Secondly, only sputum cultures were conducted upon the patients’ admission; urine and blood cultures were not performed. This limitation might have led to the underdiagnosis of some co-infections. Additionally, asymptomatic infected patients and some mild cases who declined hospitalization were not included in the study, resulting in a low hospital occupancy rate. Moreover, all the cases we analyzed were winter-onset cases, and a significant proportion of them were severe. This scenario might have contributed to higher rates of co-infections and mortality. Addressing these issues is essential to gain a deeper understanding of the complex SARS-CoV-2 pathogenesis. Finally, We have only predicted the therapeutic effects of traditional Chinese medicine and their derivatives on co-infections, and further experimental verification is necessary to understand their specific therapeutic effects.

In summary, co-infection emerges as a risk factor for COVID-19 patients, elevating the risk of severe pneumonia and mortality. Co-infection suppresses the host’s immune response by reducing the number of lymphocytes and increasing inflammation, thereby diminishing the antiviral and anti-infective effects of the immune system, which promotes the severity of the disease. Implementing infection prevention measures is crucial to mitigate COVID-19 spread among hospitalized patients. Additionally, changes in these biomarkers provide a theoretical basis for the effective treatment of co-infections with traditional Chinese medicine.

## Data availability statement

The raw data supporting the conclusions of this article will be made available by the authors, without undue reservation.

## Ethics statement

The study complied with all relevant ethical regulations and the protocol was approved by the institutional review board of the Ninth Hospital of Xi’an(NO.202311). The studies were conducted in accordance with the local legislation and institutional requirements. Written informed consent for participation was not required from the participants or the participants’ legal guardians/next of kin because Written informed consents were waived due to the very contagious nature of COVID-19. Written informed consent was obtained from the individual(s) for the publication of any potentially identifiable images or data included in this article.

## Author contributions

ZG: Data curation, Formal analysis, Funding acquisition, Investigation, Methodology, Software, Writing – original draft, Writing – review & editing. ZJ: Data curation, Formal analysis, Investigation, Methodology, Software, Writing – original draft. GQ: Data curation, Formal analysis, Investigation, Writing – original draft. ZY: Data curation, Formal analysis, Investigation, Methodology, Writing – review & editing. LY: Conceptualization, Data curation, Formal analysis, Investigation, Methodology, Project administration, Resources, Supervision, Validation, Writing – review & editing.
